# Advancing breast cancer prediction: Comparative analysis of ML models and deep learning-based multi-model ensembles on original and synthetic datasets

**DOI:** 10.1371/journal.pone.0326221

**Published:** 2025-06-18

**Authors:** Kazi Arman Ahmed, Israt Humaira, Ashiqur Rahman Khan, Md Shamim Hasan, Mukitul Islam, Anik Roy, Mehrab Karim, Mezbah Uddin, Ashique Mohammad, Md Doulotuzzaman Xames

**Affiliations:** 1 Department of Industrial and Production Engineering, Military Institute of Science and Technology, Dhaka, Bangladesh; 2 Department of Biomedical Engineering, Military Institute of Science and Technology, Dhaka, Bangladesh; 3 Department of Industrial and Production Engineering, Bangladesh University of Engineering and Technology, Dhaka, Bangladesh; 4 Department of Computer Science and Engineering, University of Dhaka, Bangladesh; Ariel University, UNITED KINGDOM OF GREAT BRITAIN AND NORTHERN IRELAND

## Abstract

Breast cancer is a significant global health concern with rising incidence and mortality rates. Current diagnostic methods face challenges, necessitating improved approaches. This study employs various machine learning (ML) algorithms, including KNN, SVM, ANN, RF, XGBoost, ensemble models, AutoML, and deep learning (DL) techniques, to enhance breast cancer diagnosis. The objective is to compare the efficiency and accuracy of these models using original and synthetic datasets, contributing to the advancement of breast cancer diagnosis. The methodology comprises three phases, each with two stages. In the first stage of each phase, stratified K-fold cross-validation was performed to train and evaluate multiple ML models. The second stage involved DL-based and AutoML-based ensemble strategies to improve prediction accuracy. In the second and third phases, synthetic data generation methods, such as Gaussian Copula and TVAE, were utilized. The KNN model outperformed others on the original dataset, while the AutoML approach using H2OXGBoost using synthetic data also showed high accuracy. These findings underscore the effectiveness of traditional ML models and AutoML in predicting breast cancer. Additionally, the study demonstrated the potential of synthetic data generation methods to improve prediction performance, aiding decision-making in the diagnosis and treatment of breast cancer.

## Introduction

Breast cancer is the most often diagnosed cancer, accounting for roughly 1 in 8 cancer cases globally, and is a significant cause of mortality for women worldwide. There were roughly 2.3 million new cases of breast cancer and 685,000 fatalities from the condition in 2020, with considerable regional and national differences [1]. Economically developing nations have greater rates of breast cancer incidence, but regrettably, they also account for a disproportionate share of breast cancer fatalities. The International Agency for Research on Cancer (IARC) and associate institutions recently conducted a study that offered a global overview of the burden of breast cancer in 2020 and anticipated its impact in 2040. By 2040, the study estimates that there will be a significant spike in both breast cancer diagnoses and fatalities, with an anticipated 40% increase in new cases annually and a 50% increase in annual deaths [1].

Efficiently and accurately diagnosing breast cancer is a major challenge in both the bioinformatics field and the medical science field [2]. The current diagnostic process benefits significantly from the expertise of medical professionals, though there are opportunities for reducing errors, minimizing biases, and streamlining procedures to enhance efficiency. Traditional methods, such as mammography, face limitations due to the vast amount of imaging data involved, which can compromise accuracy and occasionally result in misdiagnosis. Recognizing the need for improved diagnostic capabilities, ML and DL techniques have emerged as valuable tools in the healthcare industry. These advanced computational methods offer the potential for high performance in disease prediction, diagnosis, cost reduction, and real-time decision-making, ultimately aiding in saving lives [3]. Based on the outcomes of implementing several ML algorithms for breast cancer [4], heart disease [5], liver disease [6], lung cancer [7], and prostate cancer [8] prediction and notable accuracy in comparison to previous approaches, our new study aims to employ a variety of ML algorithms, including both conventional models such as Support Vector Machines (SVM), Logistic Regression (LR), K-Nearest Neighbors (KNN), Artificial Neural Networks (ANN), and Random Forests (RF) and ensemble models such as XGBoost and AutoML to address the challenges in breast cancer diagnosis. Additionally, the study will explore the potential of multi-model ensembles based on DL techniques. Furthermore, synthetic data generation models, specifically Gaussian Copula (GC) and Tabular Variational Autoencoder (TVAE) used to produce synthetic data that is derived from the original dataset. This research aims to benchmark various ML models in the detection of breast cancer by evaluating the effectiveness and accuracy of these models on both real-world and synthetic datasets.

ML techniques have become essential in various fields, including healthcare. In the context of breast cancer prediction, ML techniques play a crucial role in early diagnostics and prognosis. Shamrat et al. [9] conducted an experimental investigation using the Wisconsin breast cancer original dataset. In their study, they employed six supervised classification methods, including SVM, Naive Bayes (NB), KNN, RF, Decision Tree (DT), and LR, for early breast cancer prediction. The breast cancer dataset was scrutinized for sensitivity, specificity, F1 measure, and overall accuracy. It was discovered that the SVM recorded the top classification accuracy at 97.07%, with NB and RF following closely in second place for prediction accuracy. In 2016, Khourdifi et al. [10] carried out a study where they employed four ML techniques; these were RF, NB, SVM, and KNN. They used these algorithms on medical datasets with the aim of predicting breast cancer. The results showed that the SVM was the most accurate, achieving a high accuracy rate of 97.9%.

A study was conducted in 2020 by Deulkar et al. [11] using the Wisconsin breast cancer diagnostic dataset with the purpose of classifying data for predicting breast cancer. They used a variety of supervised ML methods, including LR, DT Classifier, RF, KNN, and SVM. From the experimental results, the RF classifier stood out, providing the highest accuracy of 96.50% in comparison to the other classifiers. The research of Guleria et al. [12] used supervised ML algorithms (KNN, NB, LR, and DT) to predict and diagnose the class of breast cancer (benign or malignant). The assessment of the classification algorithms’ performance was executed based on metrics such as accuracy, sensitivity, specificity, and the F-measure. It has been observed that the prediction model built up by naïve Bayes provides a higher accuracy (87.41%) as well as a higher F-measure (0.91) among all. Akaramuthalvi & Palaniswamy [13] analyzed and compared traditional and automated ML methodologies for breast cancer diagnosis. They utilized popular AutoML frameworks, specifically Auto-SKlearn and TPOT, to categorize breast cancer cells as malignant or benign. The results demonstrated high accuracy, with the Auto-SKlearn classifier achieving 97.5% accuracy, while the TPOT classifier scored slightly higher with an accuracy rate of 98.6%. The research of Shravya et al. (2019) [14] implemented supervised ML techniques (LR, SVM, and KNN) to predict breast cancer. It found out that SVM is the best algorithm for predictive analysis with an accuracy of 92.7% and KNN presented well next to SVM. In a study by Iparraguirre-Villanueva et al. [15], four classification methods were used to predict breast cancer. These methods are Bayes Net (BN), Adaboost, Simple Logistic, and Stochastic Gradient Descent (SGD). This research tests the accuracy, uncertainty matrix, MAE, and RMSE of each method and concludes that the Simple Logistic method is the most accurate.

Osareh et al. [16] proposed the use of ML methods like SVM, KNN, and probabilistic neural networks for the identification and diagnosis of breast cancer. Their findings revealed that SVM classifier models yielded the highest overall accuracy rates for diagnosing breast cancer, achieving impressive results of 98.80% and 96.33% accuracy respectively. In their 2018 study, Hung et al. [17] employed PySpark and its ML frameworks to construct prediction models for breast cancer. They used the Breast Cancer Coimbra Data Set which contained over a hundred data sets from regular blood tests. The resulting accuracy rates for detection and classification were approximately 72% and 83%, respectively. In their 2013 study, Ahmad et al. [18] utilized ML methods to create predictive models for recurrences in breast cancer. They compared the effectiveness of three popular algorithms and observed that the SVM classification model delivered the lowest error rate and highest accuracy, achieving a noteworthy accuracy rating of 95.7% for predicting breast cancer recurrence. DT had the lowest accuracy 93.6% for predicting breast cancer recurrence and ANN had an accuracy rating of 94.7% for predicting breast cancer recurrence.

From Table 1, we can see various research has been done in the sector of using DL for predicting breast cancer. While conducting research on this, Tiwari et al. [19] used ANN and Convolution Neural Networks (CNN) as DL models while predicting breast cancer. They found ANN and CNN with 97.3% and 99.3% accuracy respectively, which was higher than all the ML models that the author used. Mekha & Teeyasuksaet [20] in their research, compare the classification algorithms (NB, DT, SVM, Vote, RF, and AdaBoost) for breast cancer based on tumor cells. Their attention was centered on the use of DL algorithms to categorize various forms of breast cancer. By applying the Exprectifier activation function in the DL model, they discovered a high accuracy measurement of 96.99%. Zheng et al. [21] proposed a novel technique for breast cancer detection, which they called a DL-assisted efficient AdaBoost algorithm (DLA-EABA). This model achieved a high accuracy level of 97.2% while predicting breast cancer.

**Table 1:** Literature review summary.

Literature source	Technique used	Dataset	Key findings
[9]	SVM, NB, KNN, RF, DT, and LR	Wisconsin Breast Cancer Original Dataset (WBCO)	SVM obtained the highest classification accuracy of 97.07%
[10]	RF, NB, SVM, and KNN	Wisconsin Breast Cancer Original Dataset (WBCO)	SVM provides the highest accuracy at 97.9%.
[11]	LR, DT Classifier, RF, K NN, and SVM	Wisconsin Breast Cancer Diagnostic (WBCD) Dataset	RF classifier gives the highest accuracy of 96.50%
[12]	KNN, Naive Bayes, LR, and DT	UCI machine learning repository (WBCD)	The Naïve Bayes method demonstrated superior performance, yielding an accuracy rate of 87.41%.
[13]	AutoML frameworks: - Auto-SKlearn, TPOT	Wisconsin Breast Cancer (WBCD) Diagnostic Dataset	Auto-SKlearn and TPOT classifiers demonstrated remarkably high accuracy rates of 97.5% and 98.6% respectively.
[14]	LR, SVM, and KNN	UCI repository (Wisconsin Breast Cancer Diagnostic Dataset)	SVM was found for predictive analysis with an accuracy of 92.7%.
[15]	Bayes Net, Adaboost, Simple Logistic, and SGD	Wisconsin Breast Cancer Diagnostic (WBCD) Dataset	The simple Logistic method is the most accurate with an accuracy of 75.17%
[16]	SVM, KNN and PNN	Fine needle aspirate of breast lesions, Gene expression data from primary breast tumors of young patients and BRCA1 carriers.	SVM classifier models showed an overall accuracy of 98.80% and 96.33% respectively
[17]	PySpark, MLlib package from Spark, ML package from Spark and DT algorithm	The Breast Cancer Coimbra Data Set	The detection and classification accuracy rates using PySpark were approximately 72% and 83%, respectively.
[18]	DT, SVM, and ANN	ICBC dataset in the National Cancer Institute of Tehran for the years 1997–2008	SVM classification model had the highest accuracy rate of 95.7% for predicting breast cancer recurrence.
[19]	KNN, SVM, DT, Naïve Bayes, LR, RF, CNN, ANN	University of California Irvine (UCI) dataset	DL algorithms such as CNN and ANN have been implemented, with maximum obtained accuracy of 99.3% and 97.3% respectively.
[20]	Naïve Bayes, DT, SVM, RF, Adaboost, DL(Tanh), DL(Relu), DL(Maxout), DL(ELU)	Wisconsin Breast Cancer Original (WBCO) Dataset	DL by Exprectifier activation function has high accuracy of 96.99% with cross-validation.
[21]	DL Assisted Efficient AdaBoost Algorithm (DLA-EABA)	The Cancer Imaging Archive (TCIA) Public Access	The results from the experiments indicate that the suggested model attained a considerable accuracy level of 97.2%.
[22]	STACKED (RF, SVM, Naïve Bayes, LR) (Output Features), STACKED (RF, SVM, Naïve Bayes, LR) (Hidden Features)	University of Science and Technology of China’s METABRIC breast cancer dataset	STACKED RF (Hidden Features) provides the highest accuracy at 90.2%.
[23]	KNN, MLP, DT, Proposed Ensemble Model	Wisconsin Prognostic Breast Cancer (WPBC) dataset	Proposed model achieved highest average accuracy which is 96.29% than any other individual classifiers.
[24]	KNN, SVM, DT, RF, GBDT, DL-Based Multi-Model Ensemble	Dataset by The Cancer Genome Atlas Program (TCGA)	The proposed ensemble model achieved 98.41% accuracy while predicting breast cancer.
[35]	wDADA	METABRIC breast cancer dataset	The proposed method achieved 67.26% accuracy while predicting disease-specific survival (DSS).
[36]	Synthetic Data Generation: – CTGAN, TVAE ML Classifiers: - LR, MLP, KNN, SVM, XgBoost DL Classifier: - TabNet	UCI Repository Dataset (WBCD)	KNN achieved the highest accuracy (59.05%) with CTGAN-generated data, while TabNet outperformed other models with an accuracy of 96.66% when trained on TVAE-generated data.
[25]	Computational modeling, Monte Carlo optimization, simulation of socio-demographic-based SMS campaigns	Real-world U.S. socio-demographic and cancer statistics data	Optimized SMS reminder campaigns using simple socio-demographic features significantly reduce mortality rates in breast cancer patients compared to standard “one-size-fits-all” approaches.
[29]	Multidisciplinary review of epidemiologic, physiological, and molecular biology studies	No original dataset; review based on literature from MedLine, PubMed, PsycINFO (1973–2010)	Chronic stress, especially early in life, is linked to higher breast cancer risk via hormonal and immune disruption. Variability in findings highlights the need for more integrative research.
[26]	Retrospective cross-sectional analysis; logistic regression modeling	Pediatric Health Information System (PHIS) database – data from 52 U.S. tertiary pediatric hospitals	Non-white children and those with public insurance had lower CI/HA use and higher speech delay rates, revealing persistent disparities in pediatric hearing care.

The DL-Based Multi-Model Ensemble method is another efficient strategy to predict breast cancer accurately. Arya & Saha [22] constructed a stacked ensemble ML model, utilizing a Convolutional Neural Network (CNN) and a variety of ML techniques (such as SVM, RF, NB, and LR) to estimate the lifespan of patients with breast cancer. The CNN was employed for feature extraction, with the extracted features serving as inputs for the stack-based ensemble model. The STACKED RF (Hidden Features) produced an AUC score of 0.93 and an accuracy rate of 90.2% at the medium strictness level in predicting the prognosis of breast cancer. Maurya et al. [23] utilized a double RBF kernel function for feature selection and introduced a novel fusion process to improve the performance of three basic classifiers: KNN, Multi-Layer Perceptron (MLP), and DT. Their proposed model achieved commendable results, with a training accuracy of 95.83% and a testing accuracy of 96.74%, in predicting the outlook for patients with breast cancer. Xiao et al. [24] demonstrated this strategy where they applied DL to a collective approach that integrates multiple diverse ML models is being put forward. This proposed method uses DL and involves a multi-model ensemble, which is demonstrated. to be more accurate and effective than other used ML techniques (KNN, SVM, DT, RF, GBDT) for cancer prediction. This model achieved 98.41% accuracy while predicting breast cancer.

In recent years, socio-demographic factors have gained recognition not only for influencing disease risk but also for shaping patient compliance with healthcare interventions. Savchenko et al. [25] proposed a computational framework showing that personalized SMS reminders based on basic socio-demographic data (e.g., age, gender, economic status) can significantly boost check-up compliance in breast cancer patients. Their work underscores the value of integrating behavioral modeling with ML to develop context-aware, patient-centric diagnostic systems. Social determinants like race, insurance type, and economic background increasingly impact health outcomes. For example, Magee et al. [26] reported disparities in speech and hearing rehabilitation among non-white children and those with public insurance, reflecting broader issues in access to care. Similar challenges affect breast cancer prediction, where early screening and labeled data may be unequally distributed. This highlights the need for inclusive data strategies, such as synthetic data generation, and equitable machine learning models that perform well across diverse populations. Socio-demographic features—such as age, race, socioeconomic status (SES), and family history—have been consistently shown to enhance model performance when combined with clinical, genetic, or imaging data. Fo example, Feld et al. [27] reported that while demographic data alone yielded a modest predictive AUC of 0.580, integrating them with genetic and imaging features improved the model’s AUC to 0.753. First-degree family history, in particular, emerged as a significant individual predictor (p < 0.001). Dammu et al. [28] similarly observed that removing socio-demographic variables from a deep learning framework reduced the AUC for predicting pathological complete response from 0.83 to 0.67, underscoring their additive predictive value. Beyond statistical performance, these variables have operational and biological relevance. Collectively, these findings underscore the essential role of socio-demographic data in boosting model performance, ensuring fairness, and guiding real-world clinical implementation. Beyond primary physiological and genetic factors, secondary influences like psychological stress, behavior, and environmental exposures are increasingly linked to breast cancer risk and progression. Antonova et al. [29] reviewed how chronic stress and HPA axis activation—marked by elevated cortisol—can impair DNA repair, suppress apoptosis, and alter estrogen signaling, potentially impacting breast tissue biology. These insights highlight the role of lifestyle, emotional well-being, and socioeconomic status in shaping susceptibility, supporting more holistic prediction models.

Limited labeled data availability is a critical challenge in cancer diagnostic prediction, as it stems from the scarcity of labeled examples with known diagnostic outcomes. The availability and quality of labeled data play a pivotal role in developing accurate and reliable models for cancer diagnosis. However, the scarcity of labeled data poses a significant obstacle in this field [30]. Researchers have distinctly noted that the lack of breast cancer data hampers research based on artificial intelligence [31]. Thus, it is recommended that AI researchers conduct studies centered around the generation and assessment of synthetic patient data. This entails utilizing a variety of DL architectures, inclusive of cutting-edge technologies like Generative Adversarial Networks (GANs), and Variational Autoencoder (VAE) [32,33] along with statistical techniques such as the GC [34]. Hsu and Lin [35] introduced a framework known as Wasserstein GAN-based Deep Adversarial Data Augmentation (wDADA). This framework uses GANs to enhance data augmentation and support model training. The wDADA approach posted a 67.26% correctness in predicting the disease-specific survival (DSS) of breast cancer patients based on the METABRIC dataset [30]. In research led by Inan et al. [36], synthetic breast cancer data were generated through the use of the Conditional GAN (CTGAN) and TVAE. Several ML classifiers – LR, MLP, KNN, SVM, and Extreme Gradient Boosting (XgBoost) – along with a DL classifier, TabNet, were then deployed to predict cancer outcomes. Among these, KNN had the highest accuracy rate of 59.05% when using CTGAN-generated data for breast cancer diagnosis prediction. However, when using TVAE-generated data, TabNet achieved the highest accuracy rate of 96.66% for the same task [36].

Based on the literature review presented above, there are several research gaps that can be identified:

- **Exploration of AutoML Frameworks versus Traditional Approaches:** Recent research has begun to address the comparative effectiveness of AutoML frameworks versus conventional ML methodologies in predicting breast cancer [13]. However, there is still a significant gap that prompts a necessity to conduct comprehensive and comparative investigations, which scrutinize the efficacy, accuracy, and performance of AutoML frameworks like H2O, compared with traditional techniques such as SVM, K-NN, ANN, LR, and RF using the identical breast cancer dataset.
- **Evaluation of Synthetic Data Generation Techniques:** Ample data is pivotal for the seamless incorporation of DL strategies into breast cancer classification. While multiple synthetic data generation models exist, there is a deficiency of analytical work comparing the performance of these diverse synthetic data generation techniques for predictive purposes in breast cancer.
- **Comparative Study of ML and DL-based Multi-Model Ensembles on Original and Synthetic Datasets:**

The comparative performance of machine learning (ML) and deep learning (DL) models in our study aligns well with findings from recent benchmarking research on state-of-the-art (SOTA) breast cancer prediction methods. Iparraguirre-Villanueva et al. [37] conducted a comprehensive evaluation involving six ML algorithms—MLP, KNN, AdaBoost, Bagging, GB, and RF—and reported exceptionally high performance, with ensemble models achieving up to 100% accuracy on the Wisconsin Breast Cancer dataset. Similarly, La Moglia and Almustafa [38] evaluated eight classifiers and demonstrated that, following feature selection, models such as LightGBM (LGBM) and Logistic Regression achieved high accuracy levels (90.74% and 91.67%, respectively), underscoring the importance of domain-relevant features such as age, tumor size, lymph node status, and metastasis indicators. Further, Almarri et al. [39] introduced the Breast Cancer Prediction Model (BCPM) framework, which systematically applied both traditional ML algorithms and shallow neural networks to structured clinical data, significantly improving diagnostic precision. Collectively, these studies highlight that while deep learning approaches—especially those utilizing imaging data—often lead in raw prediction performance, well-tuned ML models remain highly competitive when working with curated clinical and demographic features.

Our findings support this trend: DL-based ensemble models in our study exhibited slightly higher average accuracy on both original and synthetic datasets. However, ensemble ML models such as XGBoost and RF consistently delivered strong and stable performance, particularly when supported by appropriate feature selection and hyperparameter tuning. This reinforces the utility of hybrid and interpretable models, especially in clinical settings where data may be tabular, imbalanced, or limited in volume. The existent scholarly attention towards individual ML models is extensive, but lacks comprehensive evaluation and comparison against ensemble models, as well as multi-model ensembles, specifically for both original and synthetically created datasets.

To address the research gaps identified, the current study employs a comprehensive methodology to evaluate and compare the performance of various ML models and ensemble techniques for breast cancer prediction using both original and synthetic datasets. Specifically, this research compares the capabilities of AutoML frameworks like H2O versus conventional ML approaches. Additionally, this research focuses on assessing and comparing the capabilities of data generation methods like Gaussian Copula, CTGAN, Copula GAN, and TVAE utilizing the same breast cancer dataset, measured against set evaluation metrics, and ML models. Finally, this study brings integrated deep neural network (DNN)-based multi-model ensemble along with AutoML (DL)-based multi-model ensemble into focus and compares their accuracy for original and synthetically created datasets versus an array of individual ML classifiers like SVM, RF, LR, ANN, KNN, and ensemble models such as XgBoost and AutoML. The goal is to determine the most effective techniques or combination of techniques for precise breast cancer prediction through a rigorous assessment methodology. By exploring these under-researched areas, this study aims to advance breast cancer prediction techniques.

We acknowledge that the original dataset (UCI Breast Cancer Wisconsin Diagnostic Dataset) comprises only 569 instances, it remains a well-established and extensively benchmarked dataset in breast cancer prediction research. Its widespread use enables meaningful comparisons with prior studies and supports the methodological validity of our approach. Moreover, to address limitations related to dataset size and diversity, we supplemented the original data by generating synthetic datasets using Gaussian Copula and TVAE models. These enriched datasets allowed for broader evaluation of model robustness and generalizability.

## Methods

**Fig 1:**
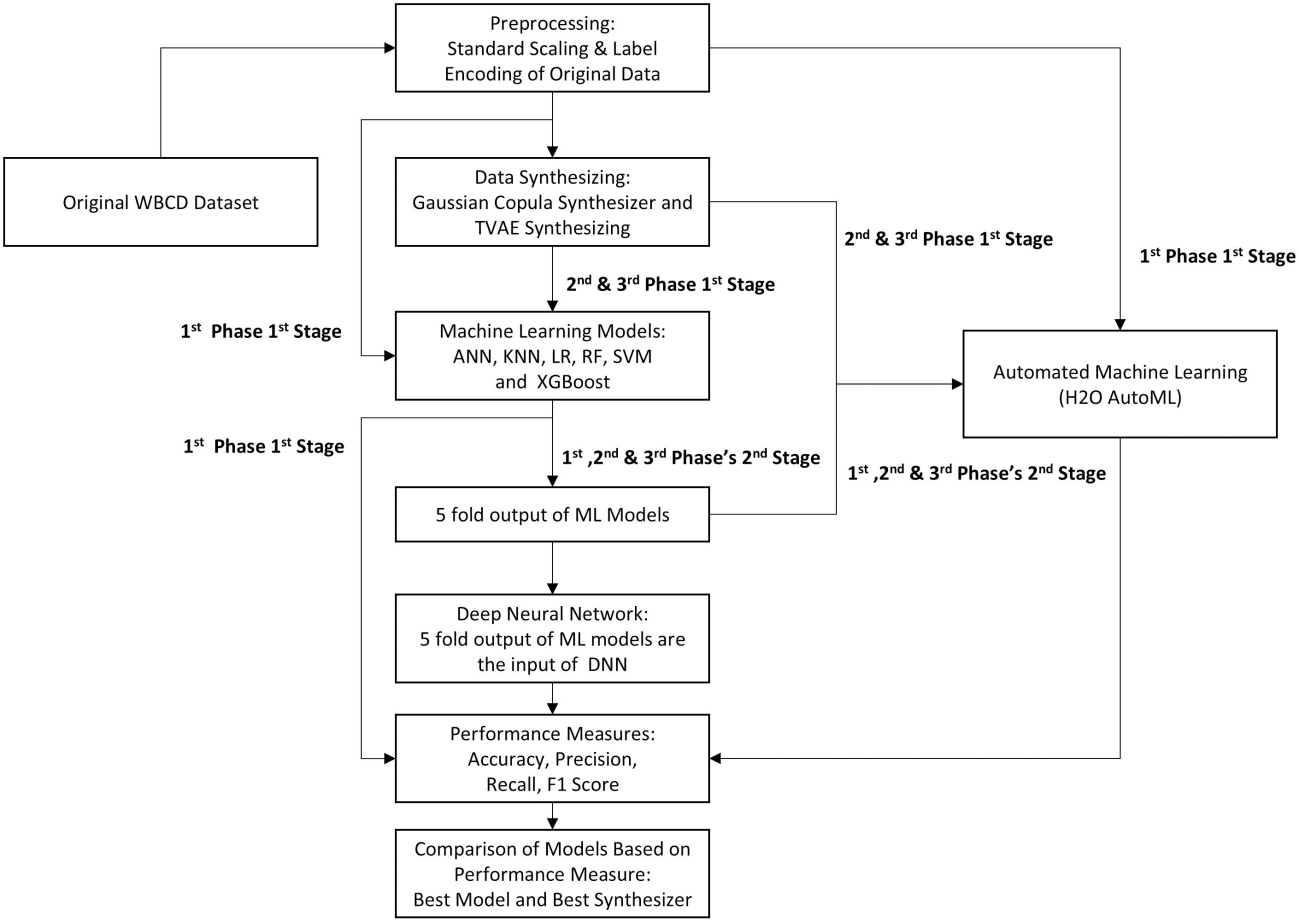
Framework of the methodology.

The purpose of this study is to assess and contrast the accuracy of various ML models, both alone and in conjunction with a DNN and an Automated Machine Learning (AutoML) strategy, for predicting breast cancer. This methodology is divided into three phases, each having two stages, to provide a complete evaluation of the model’s performance. To begin, the University of Wisconsin Madison hospitals’ breast cancer diagnostic dataset is preprocessed using conventional scaling and label encoding methods. The overarching research process for this study is depicted in Fig 1. Ethics approval was not required for this study as it used a publicly available, de-identified dataset.

As illustrated in Fig 1, In the 1st stage of the 1st phase, the stratified K-fold cross-validation technique is employed creating K groups of training and testing datasets. Multiple ML models, including KNN, SVM, ANN, RF, Xgboost, and AutoML, are sequentially trained on K-1 folds of the training set and evaluated on the corresponding test set. This process is repeatedly used for all K-folds of the dataset, and then the accuracy of each model is assessed to determine its predictive performance. In the 2nd stage of the 1st phase, a DL-based ensemble strategy [9] and an AutoML-based ensemble strategy are employed to improve the accuracy of breast cancer prediction. The predicted datasets generated by the individual ML models on the dataset, excluding predictions from AutoML, are integrated. Using these integrated datasets, an ensemble model with DNN is constructed. Additionally, the same integrated predicted datasets are used to create an ensemble model with AutoML. The accuracy of both ensemble models (DNN-based and AutoML-based) is evaluated against the corresponding test data.

In the 1^st^ stage of 2^nd^ and 3^rd^ phases, synthetic data generation techniques are employed to augment the Wisconsin Breast Cancer Diagnostic (WBCD) dataset. Gaussian Copula and Triplet-Based VAE techniques are used respectively to generate the synthetic data. These synthetic datasets are used to create stratified K-fold testing and training datasets. The subsequent steps in these phases follow a similar approach to the 1^st^ stage of the 1^st^ phase, where the stratified K-fold datasets are utilized for training and testing predefined ML models. In the 2^nd^ stage of these phases, we feed the predicted datasets (excluding AutoML’s predictions) from the ML models to the DNN and AutoML, respectively, and assess the accuracy of all individual models as well as the ensembled models with DNN and AutoML.

In the final analysis, the accuracy scores obtained from all the phases will undergo in-depth scrutiny to determine the most effective technique or combination of techniques for accurate breast cancer prediction. The primary objective of this research is to provide valuable insights into the effectiveness of different ML models, DNN, and AutoML approaches, while also investigating the influence of synthetic data for data augmentation in the context of breast cancer prediction. By evaluating and comparing the performance of these techniques, this study aims to provide a comprehensive understanding of their capabilities and potential benefits in improving breast cancer prediction accuracy.

### Cross-validation

In ML, cross-validation is a prevalent method utilized to measure and evaluate the efficiency of predictive models. The cross-validation technique entails segregating the data available into numerous subsets or folds. A segment of this data gets employed for training purposes while the rest operates for testing objectives. This procedure evaluates how competently the model can function on unfamiliar data.

The stratified K-fold cross-validation method is frequently implemented in ML. It necessitates randomly partitioning the data into equally sized sections or folds, numbered K. During each iteration, a single fold is assigned as the testing set while the rest (K-1) are used for model training. This sequence is repeated K times to ensure each fold is used for testing at least once. Through stratified K-fold cross-validation, every data point in the dataset is utilized for both training and testing, minimizing the possibility of either overfitting or underfitting the model [40]. Furthermore, by aggregating the outcomes across diverse iterations, stratified K-fold cross-validation ensures a more dependable approximation of the model’s performance. Unlike regular K-fold cross-validation, which can lead to imbalanced distributions in training and test sets, stratified K-fold cross-validation preserves the original class distribution across all folds. This ensures that each fold maintains the same proportion of each class as the entire dataset, providing a more accurate and representative assessment of model performance, particularly for imbalanced datasets. This method allows ML algorithms to be evaluated and affirmed across varied subsets inherent in the data [41]. This approach helps to assess the algorithm’s performance in various scenarios, ensuring that it can perform well on different data distributions. Overall, cross-validation plays a crucial role in ML by providing a robust method for assessing model performance and generalization ability.

**Fig 2:**
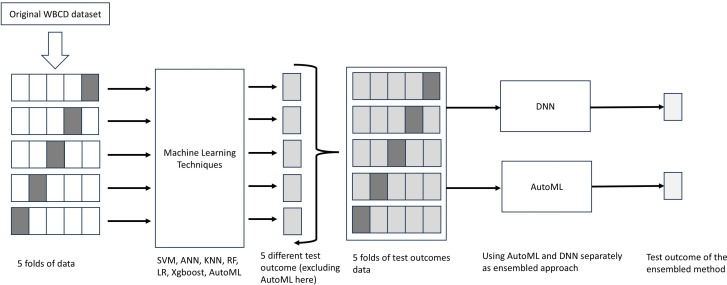
The method of stratified K-fold cross-validation and machine learning steps.

Our investigation uses a 5-fold (K = 5) stratified cross-validation approach to gauge the efficacy and generalization prowess of our ML models. As depicted in Fig 2, five subsets of equal size are produced from the original WBCD dataset via random splitting carried out by the 5-fold cross-validation. Four of these subsets are utilized for training while the remaining one serves as the test set. The averaged performance scores from these five cross-validation folds offer an accurate estimation of our model’s comprehensive performance. In our research, our use of 5-fold cross-validation aims not just to select the optimal model for each individual classifier due to variation in performance scores, but also to devise datasets in the ensemble phase that can prevent overfitting and bolster the robustness of our analysis.

### Machine learning techniques

After preprocessing the data sets, we assess the prediction performance of seven popular ML techniques toward the discrimination between normal and tumor samples. Specifically, we apply KNN, SVM, ANN, RF, LR, XGBoost, and AutoML as first-stage classification models. All seven classification methods have demonstrated high accuracy in practical applications and have been previously reviewed in the literature.

#### K-Nearest neighbor.

The KNN method is a commonly implemented ML strategy, typically categorized under instance-based or lazy learning algorithms [42]. This straightforward supervised classification algorithm assigns categories to new data points based on the classifications of its k closest neighbors within the training data. The classifier functions by identifying the k closest neighbors to a specific data point and then attributing the most frequent class label among these neighbors to it. The primary benefit of the KNN algorithm lies in its simplicity and user-friendly implementation.

If N represents the number of neighbors in the KNN technique, then the distance metric value is used to examine N samples:


$$\begin{array}{c} {Minkowski\;Distance:Dist(x,\;y)=\left(\sum {\mathrm{\backslash nolimits}}_{i=1}^{n}{\left|x_{i}-y_{i}\right|}^{t}\right)}^{\frac{1}{t}} \end{array}$$ (1)

The type of distance employed is determined by the value of t: Manhattan distance for t = 1, Euclidean distance for t = 2, and Chebyshev distance for t=∞. The most widely used of these metrics is Euclidean distance. From these K neighbors, the process determines the amount of data relevant to each class. Subsequently, the new data point is assigned to the class that constitutes the majority. This classification method is well known for its efficiency in predicting medical conditions [43–45].

#### Support vector machine.

SVM are distinguished supervised learning models often employed for classification tasks in ML. These were presented by Cortes and Vapnik [46]. SVM is a supervised learning model that aims to identify the best hyperplane in an elevated feature space to divide distinct data classes. A notable strength of the SVM algorithm is its capability to handle small sample sizes, nonlinearity, and high-dimensional pattern recognition issues [47].

The main idea behind SVM is to maximize the margin, or the distance, between the decision boundary (the hyperplane) and the nearest data points from different classes, which are known as support vectors. This emphasis on maximizing the margin allows SVM to enhance generalization and robustness during the classification process. The decision boundary of an SVM model can be represented by the equation:


$$g^{T}x+v=0$$ (2)

In this formulation, ‘g’ is the weight vector that is orthogonal to the hyperplane, ‘x’ denotes the input feature vector, and ‘v’ is the bias element. The sign of the expression $g^{T}x+v$ determines the predicted class label for a given input sample.

To train an SVM model, the optimization problem involves minimizing the objective function:


$$\begin{array}{c} min\frac{1}{2}{\left\|g\right\|}^{2}+C\Sigma \xi_{i} \end{array}$$

$$subject\;to:\;y_{i}\left(g^{T}x_{i}+v\right)\geq 1-\xi_{i},\;i=1,2,3,\ldots \ldots n$$ (3)

Where, ‖g‖ represents the Euclidean norm of the weight vector. The regularization parameter C influences the balance between achieving a wider margin and minimizing classification errors. To accommodate misclassified or margin-violating samples, slack variables $\xi_{i}\;$are introduced, while the class label for each training sample is denoted by $y_{i}$. According to Janardhana et al. [48], SVM has been identified as the most robust and effective classifier for medical datasets. Additionally, the research conducted by Vassis et al. [49] highlights the increasing utilization of SVM in medical diagnosis owing to its accurate classification characteristics.

#### Artificial neural network.

Inspired by the structuring and functionalism of biological neural networks, ANNs manifest as a highly potent technique within the ML framework. By utilizing interlinked “neurons” or nodes, ANNs excel at identifying intricate patterns within data and conducting thorough analyses. Their capacity to establish sophisticated relationships and render precise predictions has brought them under the spotlight in many different domains. A significant investigation led by Rumelhart et al. [50] set the groundwork for comprehension of the fundamental aspects of ANNs along with their associated learning algorithms. The scholarly team presented the backpropagation algorithm which empowers ANNs to refine the weightage of links between neurons and improve the network’s effectiveness. The widespread application of this algorithm has played a pivotal role in the successful training and deployment of ANNs.

The mathematical representation of an ANN model can be expressed with *n* input neurons ($x_{1},x_{2},x_{3}\ldots ,x_{n}$), *h* hidden neurons ($z_{1},z_{2},z_{3}\ldots ,z_{n}$), and *m* output neurons ($y_{1},y_{2},y_{3}\ldots ,y_{n}$) as follows:


$$y_{k}=g_{A}{\left(\sum z_{j}b_{jk}+b_{k}\right)}_{\cdots }(\;j=1-h\;)$$ (4)

In which,


$$Z_{j}=f_{A}{\left(\sum x_{i}w_{ij}+t_{j}\right)}_{\cdots }(\;i=1-n\;)$$ (5)

Where, $t_{j}\;$ represents the bias for neuron $z_{j}$ and $f_{k}$ is indicative of neuron $y_{k}$. The weight of the connection transitioning from neuron $x_{i}$ to $z_{j}$ is denoted by $w_{ij}$, while beta denotes the weight of the connection from neuron $z_{j}$ to $y_{k}$. The activation functions are represented by $g_{A}$ and $f_{A}$ respectively [51]. This formula illustrates the technique by which ANNs calculate the weighted aggregation of inputs, execute an activation function, and generate an output. Through iterative training that modifies weights and biases, ANNs have the capability to come close to approximating complex functions, thereby making predictions on data that has not been previously seen. Azar et al. [52] noted the successful utilization of ANNs in in many fields of clinical medicine areas to address complex and disordered issues, without requiring mathematical models or a precise understanding of the involved processes.

#### Random forest.

RF is a well-regarded ML method and a powerful tool for classification and regression tasks. It is recognized for its precision and resilience [53]. RF is a robust ML strategy that employs multiple decision trees to generate predictions. Each decision tree in the forest is formulated with a subset of the input features and a random selection of the training instances. The ultimate prediction is deduced by collating the predictions of every individual tree within the forest. The fundamental research introducing the RF approach was carried out by Breiman [54], who underlined the benefits of applying randomness and ensemble methodologies to enhance the precision and stability of decision trees. Research conducted by Subhapriya et al. [55] in the domain of medical science demonstrates that the RF algorithm is capable of generating precise predictions of patient outcomes from a substantial volume of data.

In the RF method, decisions from multiple DT are unified, which cancels out potential overfitting and improves the model’s ability to generalize. The process of this method can be summarized as follows:

i Choose D data samples randomly from the training set.
ii Construct a DT using these D chosen samples.
iii Decide on the number of N-trees and replicate steps 1 and 2.
iv Build an N-tree to predict the class relevant to the data samples for fresh data point and assign the new data point to the class that shows the highest probability.

#### Logistic regression.

LR has gained substantial recognition as a pervasive statistical method for classification challenges, which require predicting binary or categorical results based on input features or variables [56]. This method operates by drawing an association between the independent variables and the probability of the output falling into a certain category. As a result, the output is expressed as a binary variable with two possible outcomes. Its primary use is to predict a binary outcome (for instance, 1/0, Yes/No) relying on a set of predictor variables. Instead of creating a direct line-fit to this binary outcome, LR incorporates a transformation of the outcome known as a logit or log odds. This logit is intrinsically associated with the likelihood of the outcome. However, while probabilities are restricted to lie between 0 and 1, logits can range from minus infinity to plus infinity. The connection between the logit and the probability (P) is accordingly:


$$\begin{array}{c} \text{logit}=\ln\left(\frac{p}{1-p}\right) \end{array}$$ (6)

In LR model, the linear combination of input features is calculated as:


$$x=\alpha_{0}+\alpha_{1}x_{1}+\alpha_{2}x_{2}+\ldots +\alpha_{p}x_{p}$$ (7)

where, x is the linear combination of the input features and their respective weights. α₀, α ₁, α₂,..., α_p_ are the coefficients or weights assigned to each input feature, x₁, x₂,..., x_p_ are the values of the input features. The coefficients α₀, α ₁, α₂,..., α_p_ are estimated during the training process using optimization techniques like maximum likelihood estimation.


$$\begin{array}{c} \text{The logistic function can be expressed as: }P(x)=\frac{{𝕖}^{x}}{1+{𝕖}^{x}} \end{array}$$ (8)

where, P(x) represents the predicted probability that the outcome variable (y) takes on a specific value. This function is also known as the sigmoid function. Panda et al. [57] conducted a review on the applicability of LR in medical decision-making, concluding that it presents an efficient approach for healthcare researchers in making decisions via predictive modeling. Similarly, Mothukuri et al. [58] described the effectiveness of the LR system specifically in the prediction of heart disease.

#### Extreme gradient boosting decision tree.

Extreme Gradient Boosting Decision Trees (XGBoost) is a robust ML method that has increasingly garnered attention in recent times. Initially proposed by Chen and Guestrin [59], it was presented as a scalable solution for tree boosting. XGBoost is a widely utilized method for both classification and regression tasks, well-known for its capacity to handle sophisticated datasets with excellent precision. It serves as an optimized version of gradient boosting, an ML ensemble technique that amalgamates weak learning models, typically decision trees, to construct a robust predictive model. The model operates in an additive manner, with new weak learners added sequentially to the ensemble, each striving to rectify the mishaps of their predecessors. The final prediction is an aggregation of the predictions from all weak learners. The whole process is overseen by the gradient descent optimization that seeks to minimize a particular loss function. The prediction is derived from the summation of all individual weak learners’ predictions, with each prediction weighed by their corresponding learning rates (β):


$$F(x)=\sum \beta_{i}*f_{i}(x)$$ (9)

In this formula, F(x) signifies the ultimate prediction, βᵢ denotes the learning rate of the i-th weak learner, and fᵢ(x) showcases the prediction made by the identical weak learner. The learning rates manage the magnitude of each weak learner’s influence on the final prediction. allowing for fine-tuning of the model’s overall behavior. The XGBoost algorithm incorporates several key components to enhance model performance. It introduces regularization techniques such as shrinkage (learning rate) and column subsampling (feature subsampling) to prevent overfitting. Additionally, XGBoost employs a novel technique called “gradient boosting with approximate greedy algorithm” to efficiently construct DT, resulting in improved computational efficiency. When predicting medical interventions for patients suffering from acute bronchiolitis, Mateo et al. [60] discovered that the XGBoost method delivered superior prediction accuracy compared to other supervised learning methods. Murty et al. [61] determined that, when accurately predicting liver disease, the XGBoost model demonstrated superior classification accuracy compared to any other models currently established by ML researchers.

#### Automated machine learning.

AutoML represents an advanced machine learning approach designed to streamline the process of model creation and selection by automating multiple tasks into a unified workflow [62]. AutoML algorithms leverage sophisticated optimization techniques to explore a wide range of machine learning models and their corresponding hyperparameters. The primary goal is to identify the optimal model configuration that maximizes a specific performance metric, such as accuracy or Area Under the Curve (AUC). This automation minimizes the need for manual trial-and-error processes, reducing human bias and providing a standardized and efficient pipeline for model development. AutoML has demonstrated potential in assisting medical practitioners by uncovering novel insights and biomarkers for diseases [63].

One prominent AutoML framework is H2O, which offers a powerful suite of tools tailored for large-scale machine learning tasks. While dense neural networks (DNNs) are most appropriate for structured tabular data, H2O’s AutoML goes beyond manually tuning a single model. It systematically explores a broader range of hyperparameters and combines multiple architectures through ensemble techniques, such as gradient boosting machines, random forests, generalized linear models, and deep learning algorithms. This comprehensive optimization process results in improved model performance, scalability, and robustness. AutoML’s automated pipeline ensures efficiency and consistency, enabling practitioners to achieve superior results compared to traditional manual tuning. In its existing form, this framework contributes significantly to quick prototyping and has the potential to reduce the length of development and deployment cycles [64]. In the second stage of our investigation, we employed the DL modules of H2O to develop a fresh ensembled technique and found that this model wasn’t appropriate here as the dataset is not spatial or temporal. We then compared its predictive accuracy against the ensemble model with DNN.

### Deep learning-based multi-model ensemble

Various classification models have been proposed for predicting breast cancer, yet reaching absolute accuracy remains a challenge due to inherent limitations and errors in different aspects of each model. To mitigate this, the practice of a multi-model ensemble has emerged as an effective strategy. This approach utilizes the predictions of numerous classifiers as inputs for a subsequent learning model. This follow-up model is trained to perfect the amalgamation of predictions derived from initial models, thereby producing a final series of predictions. Given its ability to harness the advantages of different models and adequately combine their predictions, this multi-model ensemble technique possesses the potential to enhance the performance in predicting breast cancer.

In this study, during the second stage of all the phases, we first selected the proposed ensemble model by Xiao et al. where he utilized a DNN as the ensemble method to integrate multiple classification models [24]. A five-layer neural network has been used to optimize the combinations of different classifier predictions. The output layer of this network consists of only one neuron which is either 0 or 1, denoting benign or malign, respectively. To compare the prediction accuracy of Xiao et al.’s [24] model, we developed another multi-model ensemble where we used AutoML instead of DNN. AutoML is used to optimize the combination of predictions found from the first stage and generate the final set of predictions.

**Fig 3:**
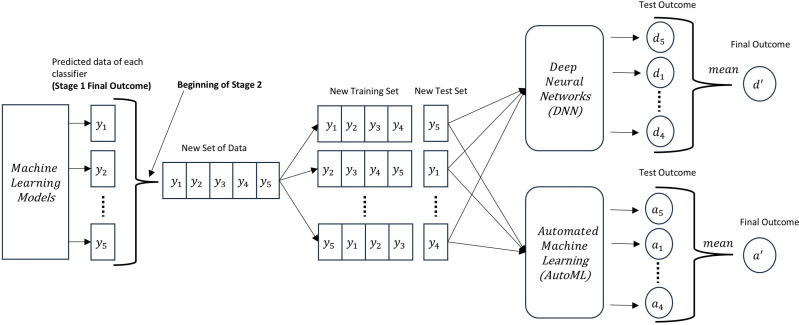
The steps in Stage 2.

Fig 3 illustrates a two-stage ensemble modeling process that begins by utilizing five different ML models to predict outcomes across the dataset using 5-fold cross-validation. As we are using 5-fold cross-validation, 5 sets $(y_{1},y_{2}\ldots ,y_{5}\backslash )$of test outcome is generated from each classifier collectively representing predictions for the entire dataset. In the second stage, these predicted outputs are consolidated into a new dataset, which is then divided into a new training set and test set. This newly formed dataset is fed into two advanced ensemble approaches: a Deep Neural Network (DNN) and an Automated Machine Learning (AutoML) framework. Both models are trained on the new training set and evaluated on the new test set to produce final test outcomes. Here, test outcomes for DNN are denoted by $d_{1},d_{2},d_{3},d_{4},d_{5}$ and the final outcome is $d^{\prime }$. Similarly, test outcomes of AutoML are $a_{1},a_{2},a_{3},a_{4},a_{5}$ and the final outcome is $a^{\prime }.$

### Synthetic tabular data generation

One of the challenges in predicting medical conditions using ML techniques is the limited availability of real medical data [65]. ML models learn patterns and make predictions based on the patterns present in the data they are trained on. When the dataset is large, it provides more diverse and representative samples, enabling the models to capture complex patterns and generalize well to unseen data. This issue can be addressed by using synthetic data generation techniques to augment the existing dataset and create new samples for training the ML model [66]. It creates artificial data points that mimic the characteristics of real-world medical data. Having a large dataset offers several benefits in ML. Firstly, it helps in reducing the impact of sampling bias, where the training data may not accurately represent the overall population. Secondly, large datasets allow for more accurate estimation of model parameters. With more data points, statistical estimates become more stable and precise, reducing the chances of overfitting or underfitting the model. This leads to improved model performance and generalization to unseen data.

**Fig 4:**
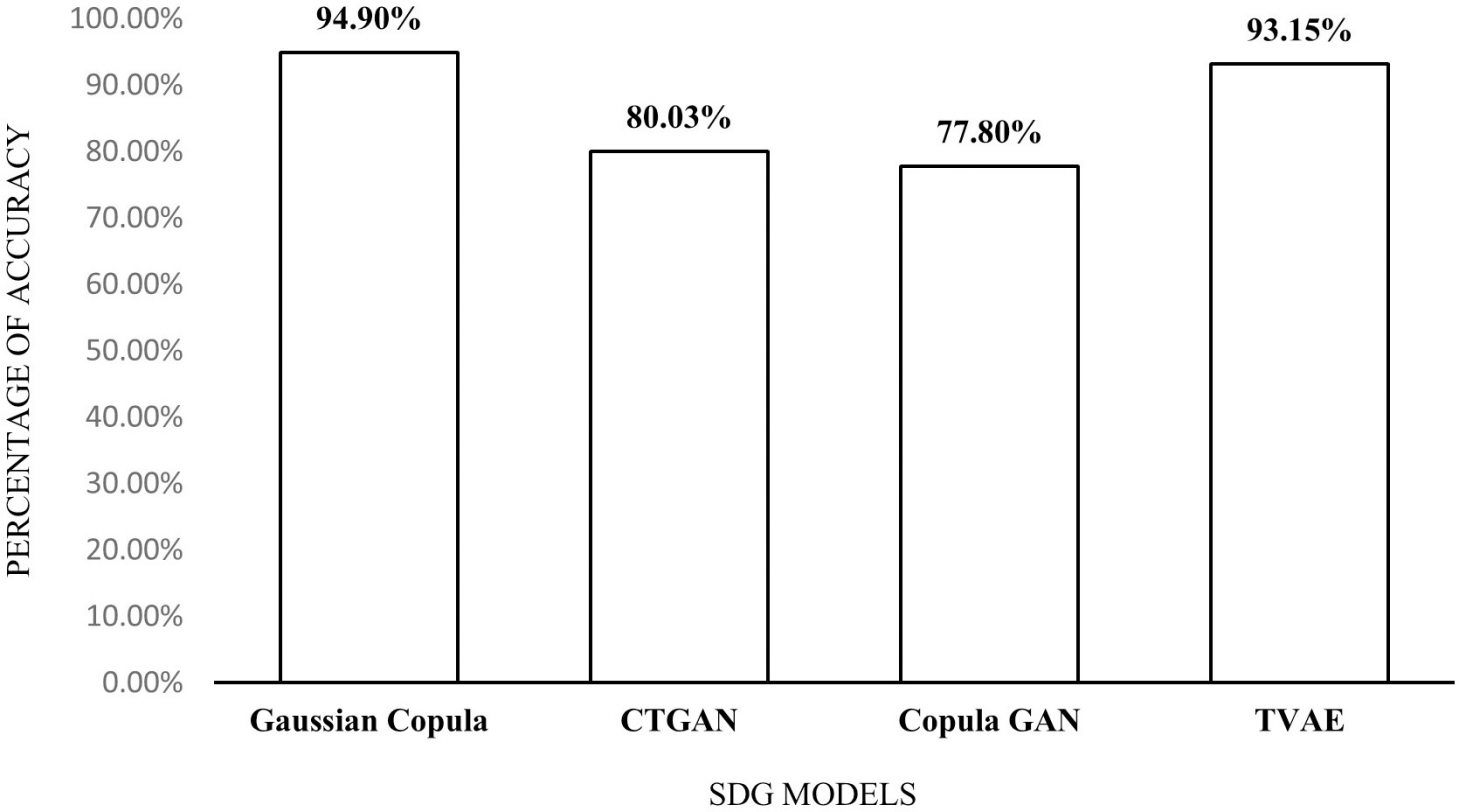
Percentage of accuracy scores for 10000 synthetic data generation.

In our study, we created 10,000 synthetic data of the original WBCD dataset using Gaussian Copula, Conditional Tabular GAN (CTGAN), Copula GAN, and TVAE. As shown in Fig 4, we found synthetic Gaussian Copula and TVAE with more than 90% accuracy score. A Python module called sdv, developed by MIT Data to AI Lab [67], is used to generate synthetic data for those synthetic data generation (SDG) techniques.

We acknowledge that synthetic datasets may not fully capture the complexity and heterogeneity present in real-world clinical data. The underlying generative processes often struggle to replicate rare edge cases, subtle feature interactions, or noise patterns typical in actual medical datasets. However, synthetic data offers a compelling privacy-utility trade-off, especially in sensitive domains like healthcare where data sharing is restricted. By generating data that preserves statistical distributions and inter-variable relationships without revealing identifiable patient records, synthetic methods like TVAE help address privacy concerns while maintaining high model utility. As shown in our results, the distribution of benign vs. malignant samples in the synthetic datasets (e.g., ~ 60% benign in TVAE vs. 62.7% in real data) demonstrates reasonable alignment with the original dataset. Nonetheless, further validation on external real-world datasets is necessary to assess generalization capabilities. Future work will also investigate integrating differential privacy techniques with generative models to strengthen both data utility and privacy guarantees.

#### Gaussian copula.

Gaussian Copula Synthetic Data Generation is a powerful tool used to capture complex dependency structures between variables [68]. A Gaussian copula is a statistical method used to characterize a selection of variables as a multivariate Gaussian distribution across m-dimensions. It offers an advantage over the conventional multivariate normal distribution as it does not necessitate the initial normal distribution of each variable [69]. The Gaussian Copula generates synthetic data by capturing the correlation structure between variables, allowing for the generation of realistic datasets. The synthetic data generated using Gaussian Copula can be utilized alongside real data to enhance the training process and improve the performance of ML models.

The objective of the Gaussian Copula is to model the joint distribution of two random variables using copula functions. Suppose we have Φ representing the standard normal distribution function, and X and Y as standard normal random variables. If X and Y have a bivariate normal distribution with a correlation of ρ, then the joint distribution of ϕ(X) and ϕ(Y), termed the Gaussian copula, depicts the relationship between these variables. The Gaussian copula C is referred as the joint distribution of ϕ(X) and ϕ(Y) by,


C(x,y)=p(ϕ(X)≤x, ϕ(Y)≤y) (10)

Gaussian Copula is a suitable method for generating synthetic data from the WBCD dataset due to its ability to capture the joint dependence structure between variables. By fitting a Gaussian Copula model to the original data, the model can learn and replicate the underlying relationships among features relevant to breast cancer diagnosis. This allows for the generation of synthetic data points that closely resemble real-world instances, preserving the correlation and dependence patterns observed in the original dataset. In our study, we used Gaussian Copula at the beginning of the 2^nd^ phase to augment the original dataset.

#### Variational autoencoder.

VAE are a type of generative model that can be used to learn the underlying distribution of a given dataset. It can learn the distribution of latent variables and reconstruct data. These latent variables capture the important features and patterns in the data, allowing for more accurate predictions. VAE consists of two main elements: a decoder and an encoder. The encoder network takes an input data point and maps it to latent variables. The decoder network then takes a sample from the latent variables and reconstructs the original input data. The key innovation of VAE lies in its probabilistic framework and the use of a variational inference technique to learn the latent space. Instead of directly learning the latent variables, VAE introduces a probabilistic distribution, typically a Gaussian distribution, to model the latent space. The encoder network learns to parameterize this distribution, mapping the input data to the variance and mean of the latent distribution. In their latest research, Xu et al. [70] employed a VAE specifically designed for tabular data, referred as the TVAE. This study utilized the TVAE to generate synthetic breast cancer diagnostic data. The TVAE model, through the implementation of Evidence Lower Bound Loss (ELBO), effectively handles the sensitivity inherent in breast cancer patient data. The ELBO is a key concept in Variational Inference, and it is the objective function that is maximized when training a VAE. The ELBO is given by the equation:


ELBO=E[logp(x|z)]−KL(q(z|x)||p(z)) (11)

E[logp(x|z)] is the expected reconstruction error, computed as the log-likelihood expectation of the data under the decoder distribution, where the expectation is taken over the encoder distribution. KL(q(z|x)||p(z)) is the Kullback-Leibler (KL) divergence between the encoder distribution (q(z|x) and the prior p(z) on the latent variables. The KL divergence measures the dissimilarity between two probability distributions. The goal is to maximize the ELBO, which is equivalent to minimizing the difference between the log-likelihood of the data and the KL divergence term. This encourages the encoder’s distribution to be similar to the prior, while also encouraging the decoder to accurately reproduce the data. TVAE is proficient in learning the covert representation of data and creating new samples mirroring the original dataset’s attributes. This capacity allows to produce synthetic data points that maintain the statistical properties and patterns inherent in the original WBCD dataset. Therefore, it’s evident that TVAE is a highly useful tool for various tasks, such as boosting data volume, ensuring privacy while sharing data, and performing sensitivity analysis, especially in the field of breast cancer diagnosis. In our study, we used TVAE at the beginning of the 3^rd^ phase to augment the original dataset.

## Results

The following section offers a comprehensive presentation of the experimental results, confirming the outcomes of each phase of this study. The experimental setups and their corresponding outcomes are meticulously described to validate the findings of this research.

### Data set description and preprocessing

We evaluated the proposed methods on the Wisconsin Breast Cancer Diagnostic (WBCD) dataset, which was collected from the popular UCI Machine Learning repository [71]. This denotes characteristics derived from digitized pictures of fine needle aspirates (FNA), which are samples extracted from breast lumps. The dataset contains a total of 569 instances, with 212 labeled as malignant (indicating the presence of breast cancer) and 357 labeled as benign (indicating non-cancerous cases). Each instance is described by 31 numerical features, including attributes such as radius, texture, smoothness, compactness, symmetry, and fractal dimension. These features capture various characteristics of the breast mass, which can be informative for predicting the presence or absence of breast cancer. We used Gaussian Copula and TVAE to generate 10,000 synthetic data.

From Table 2, we can see Gaussian Copula generated 6085 data with benign tumor which is 60.85% of total amount of generated synthetic data and TVAE generated 5974 data with benign tumor which is 59.74%. For the original data percentage of benign tumor was 62.74%. The generated synthetic data created overall 60% benign tumor data against the full dataset. During the preprocessing of the data, we removed “id” column from the dataset as it is not a significant feature to consider. Then, the categorical target variable “diagnosis” is encoded using a label encoder to convert it into numerical values. At last, to ensure that feature values fall within similar scales and ranges, they undergo standardization. This process involves deducting the mean from each feature value and then dividing it by the standard deviation. This standardization process helps to prevent any particular feature from dominating the model based on its scale, allowing for a more balanced and accurate learning process.

**Table 2:** Data sets information.

Samples		Dataset	
	Original	Gaussian Copula	TVAE
Benign	357	6085	5974
Malignant	212	3915	4026
Total	569	10000	10000

### Hyper-parameter settings

To achieve optimized hyperparameters and high accuracy, Grid Search (GS) was employed as the hyperparameter optimization algorithm for the machine learning (ML) models. Despite its exhaustive search approach, GS is widely used in healthcare applications due to its simplicity and ease of implementation. To reduce computational costs, certain parameters were fixed while others were selected for exploration using GS. By narrowing the search to a subset of critical hyperparameters, the computational burden was minimized, striking a balance between thoroughness and practicality. This strategy ensures that essential parameters are optimized without excessively expanding the search space. As a result, GS remains an effective tool for identifying optimal configurations, particularly when combined with a focused parameter selection approach.

Table 3 presents the hyperparameters used in this study, which were consistent across all three datasets. Hyperparameter settings refer to the predetermined values assigned to the hyperparameters of an ML algorithm. These user-defined parameters play a crucial role in influencing the algorithm’s behavior and performance during model training. Selecting appropriate hyperparameter settings is vital for achieving optimal model performance and often involves experimentation and fine-tuning [72].

**Table 3:** Hyperparameters for all models for three datasets.

Classifier	Hyperparameter for all Three Datasets
ANN	Layer 1: Neuron 30, activation = “relu”, Layer 2: Neuron 10, activation = “relu”, Layer 3: Neuron 01, activation = “sigmoid”, Optimizer = “adam”, loss = “BinaryCrossentropy”
DNN	Layer 1: Neuron 30, activation = “relu”, Layer 2: Neuron 25, activation = “relu”, Layer 3: Neuron 20, activation = “relu”, Layer 4: Neuron 15, activation = “relu”, Layer 5: Neuron 10, activation = “relu”, Layer 6: Neuron 05, activation = “relu”, Layer 7: Neuron 02, activation = “relu”, Layer 8: Neuron 01, activation = “sigmoid”, Optimizer = “adam”, loss = “BinaryCrossentropy”
KNN	n_neighbors = 3, 5 and 11 ^***^
LR	C = 0.1, solver = ‘liblinear’, max_iter = 1000, penalty=’l1’,
RF	n_estimators = 500, max_features = ‘sqrt’, max_samples = 100
SVM	C = 0.1, gamma = “auto” & kernel = “RBF”
XGBoost	Objective = ‘binary:logistic’, max_depth = 6, **Original Dataset:** Alpha = 10, learning_rate = 0.03, n_estimators = 250 **GC & TVAE:** Alpha = 50, learning_rate = 0.01, n_estimators = 250
H2O AutoML	max_models = 10, nfolds = 10, sort_metric = ‘accuracy’

*** For Original, TVAE and GC the hyperparameter “n_neighbours” is set to 3, 5 & 11 respectively.

Hyperparameters guide the learning algorithm by influencing updates to the model’s internal parameters during training. The chosen values directly impact model performance, requiring a careful trade-off between complexity and generalization ability. By systematically optimizing these settings, the study ensured that the models achieved high accuracy and reliable results while maintaining computational efficiency. We kept the hyperparameters the same for all three datasets to manage the computational demands of grid search. As an exhaustive search algorithm, grid search is computationally intensive and time-consuming, especially when applied to large datasets.

The Table 3 summarizes the hyperparameter settings for various classifiers applied to three datasets. The hyperparameters are specific configurations that define the behavior and performance of each classifier. The chosen hyperparameter values, such as the number of neurons, activation functions, optimizers, and loss functions, impact the model’s performance and guide the updates to the internal parameters. For instance, ANN and DNN have multiple layers with varying numbers of neurons and activation functions. LR, SVM, and RF have parameters like regularization strength, kernel type, and number of estimators. XGBoost utilizes parameters related to tree depth, learning rate, and objective function. H2O AutoML incorporates settings such as the maximum number of models, number of folds, and sorting metric for evaluation. Here we used Optimized hyperparameters to enhance classifier performance and achieve accurate predictions on the given datasets.

#### Evaluation of Phase 1: Prediction model based on the original WBCD dataset.

Table 4 presents the performance measures of different ML models trained on the original WBCD dataset. The models evaluated include ANN, DNN, KNN, LR, RF, SVC, XGBoost, H2OXGBoost, and H2ODeepLearning.

**Table 4:** Performance measures for ML models of original dataset.

	train	test	SCVTrain	SCVTest	Precision	Recall	F1_Score	Time
ANN	0.964835	0.929825	0.950786	0.954339	0.924603	0.924603	0.924603	5.586303
DNN	0.626374	0.622807	0.620614	0.619469	0.316964	0.486301	0.383784	74.24432
**KNN**	**0.982418**	**0.973684**	**0.980263**	**0.982301**	**0.974106**	**0.969246**	**0.971583**	**0.000888**
LR	0.984615	0.973684	0.980263	0.964602	0.969702	0.974206	0.971863	0.005752
RF	0.975824	0.947368	0.973684	0.964602	0.938299	0.953373	0.944481	0.8455
SVM	0.953846	0.947368	0.952551	0.952585	0.94026	0.948413	0.94399	0.039052
XGB	**0.991209**	0.947368	0.993421	0.99115	0.94026	0.948413	0.94399	2.533981
H2OXGBoost	0.973626	0.964912	0.973626	0.964912	0.958074	0.967257	0.962302	63.03487
H2ODL^*^	0.637363	0.631579	0.637363	0.640351	0.691589	0.547945	0.364527	831.7424

* DL-based Multi Model Ensemble

**Fig 5:**
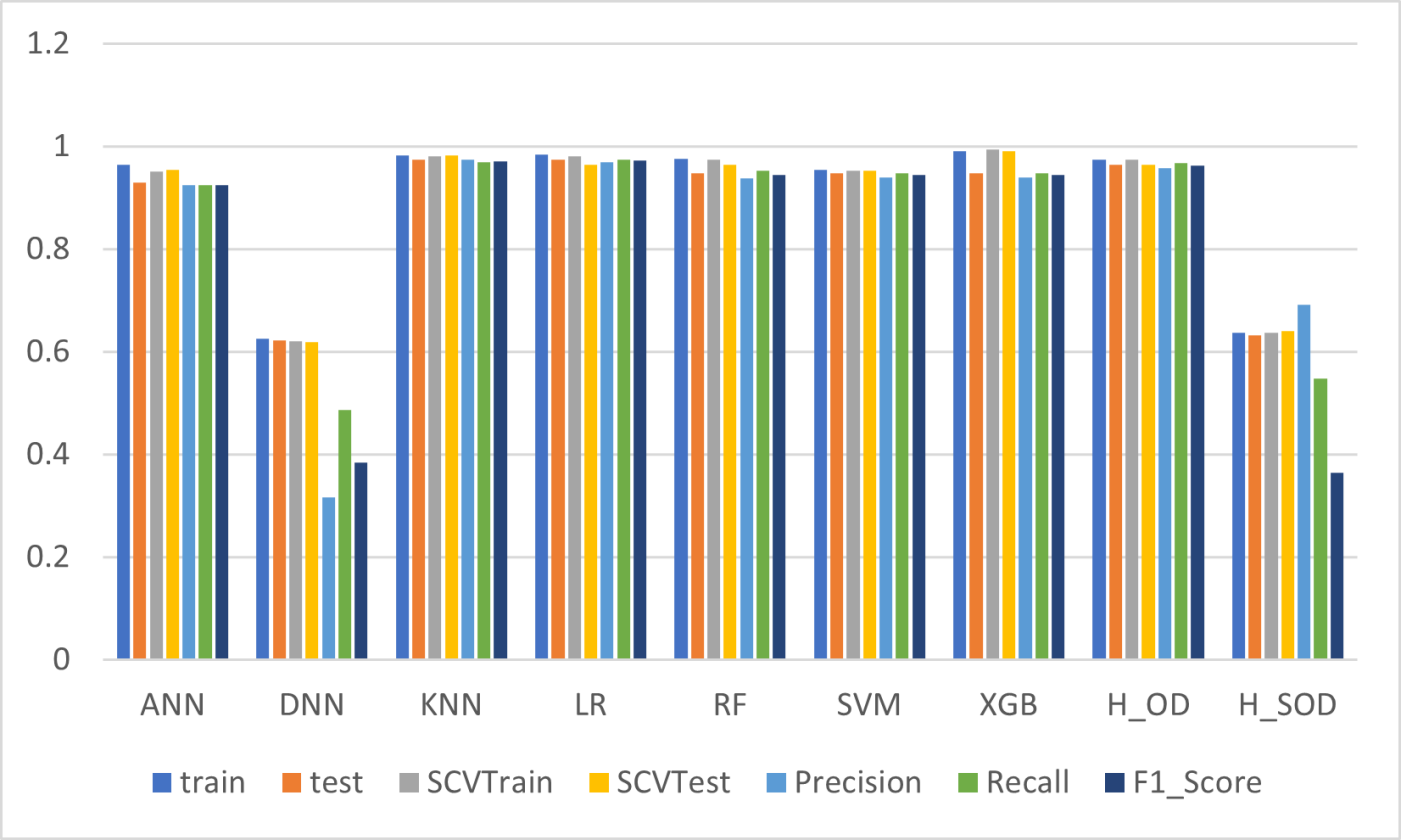
Comparison of Model Performance Metrics of original data.

In Table 4, among the evaluated models, **KNN** stands out as a consistently high performer, achieving the highest training accuracy (98.24%) and a strong test accuracy (97.37%). It also maintained impressive stratified cross-validation (SCV) scores, with 98.03% for training and 98.23% for testing, reflecting its robustness across different data partitions. Additionally, KNN achieved excellent precision (97.41%), recall (96.92%), and F1-score (97.16%), indicating its ability to accurately classify instances within the WBCD dataset. Notably, it also exhibited the fastest processing time (0.0009 seconds), underscoring its computational efficiency. In contrast, the **LR** model also performed exceptionally well, with a training accuracy of 98.46% and a test accuracy of 97.37%, closely matching KNN in overall effectiveness. However, its slightly lower SCV test score (96.46%) suggests a minor reduction in consistency across different data splits. The **RF** and **XGB** models also demonstrated strong classification capabilities, achieving high precision and recall, but with notably longer processing times compared to KNN. In below we can see the visualization of the result in Fig 5. It provides a visual comparison of their training and test accuracies, precision, recall, F1_score, and training time. The graph helps to identify the top-performing models and their relative strengths, aiding in the selection of the most suitable model for the dataset.

**Fig 6:**
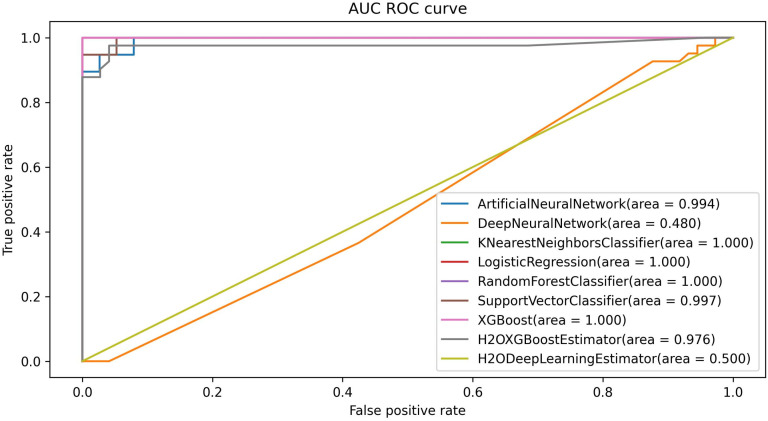
AUC ROC curves of all models for original dataset.

The Area Under the Curve (AUC) and Receiver Operating Characteristic (ROC) curves are critical for assessing the classification performance of the models. A higher AUC score indicates a better ability to distinguish between the classes, reflecting the model’s overall discriminatory power. In Fig 6, the ranking of the models based on their AUC values is as follows: KNN, LR, RF, and XGB, all achieving a perfect AUC of 1.000, indicating exceptional classification capabilities. The SVM also performed well with an AUC of 0.997, demonstrating strong discrimination despite a slightly lower score. In contrast, the ANN achieved an AUC of 0.999, placing it just below the top-tier models but still reflecting robust performance. However, the H2O XGBoost had a slightly lower AUC of 0.976, while the H2O DeepLearning and DNN exhibited significantly lower AUCs of 0.506 and 0.473 respectively, indicating weaker classification performance. These results highlight the superior classification capability of KNN, LR, RF, and XGB, making them more reliable for this dataset. The lower AUC values for the deep learning models (H2O DeepLearning and DNN) suggest the need for further tuning or alternative architectures to enhance their predictive power.

#### Evaluation of Phase 2: Prediction model based on the synthetic data created by Gaussian Copula model.

**Fig 7:**
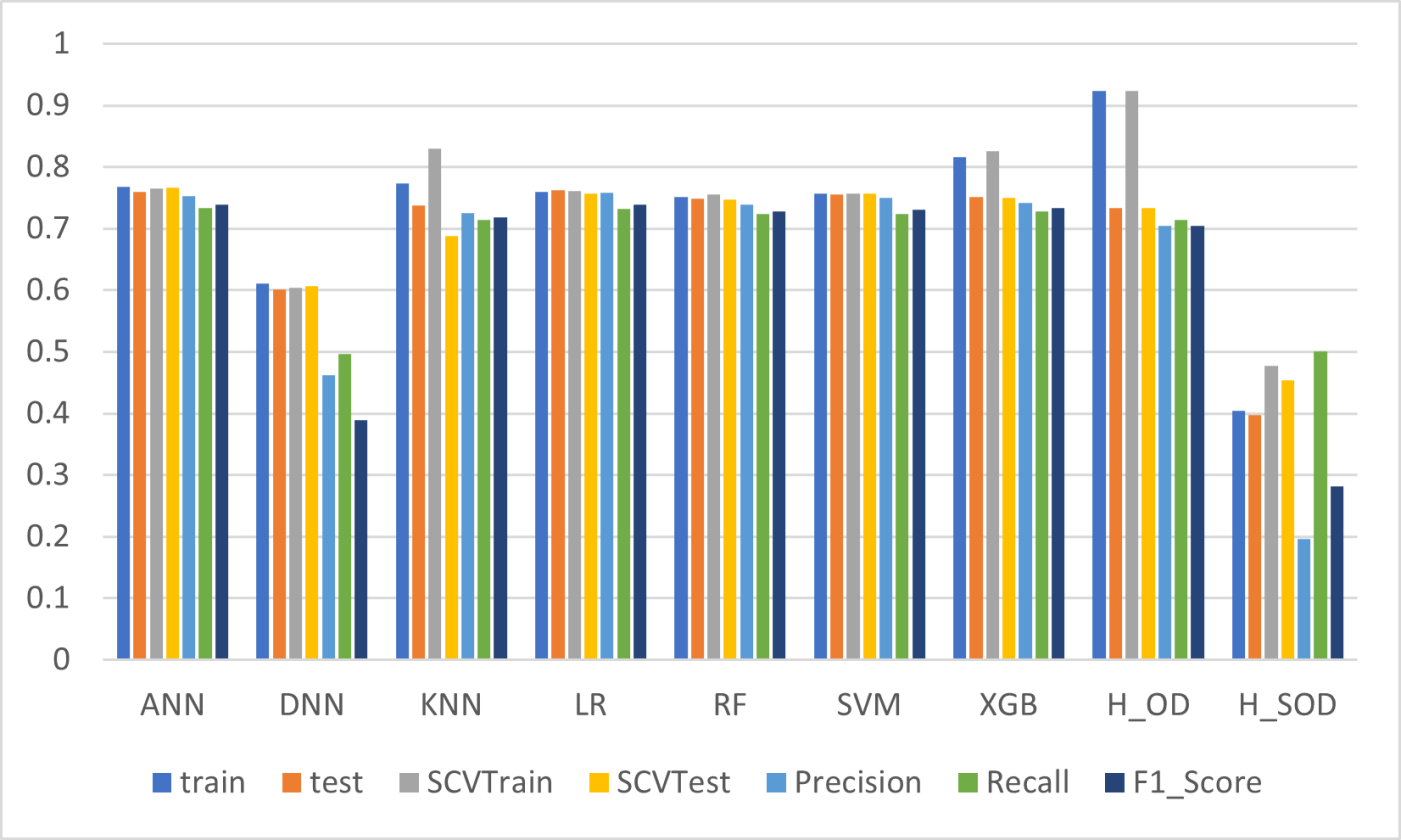
Comparison of Model Performance Metrics of GC data.

Table 5 presents the performance metrics of various ML models applied to a dataset utilizing GC synthetic data. For the evaluation, the test set used in these comparisons was retained from the original dataset, ensuring a fair assessment of the predictive performance. This setup allows for a direct comparison of models trained on real data versus those trained on synthetic data, with the testing conducted on original data. To keep the fairness for all the models, the test set from original real data was used to calculate the testing accuracy for all models on each dataset. Among the models, H2OXGBoost (H_OD) demonstrated the highest training accuracy of 0.9231, reflecting strong learning from the training data. However, this model exhibited significant overfitting, as indicated by the large gap between its training accuracy (0.9231) and testing accuracy (0.7330), suggesting a reduced ability to generalize effectively to unseen data. In contrast, XGB emerged as a more balanced performer, achieving a respectable training accuracy of 0.8161 and a testing accuracy of 0.7515. It also maintained competitive values across other key metrics, including precision (0.7411), recall (0.7284), and F1-score (0.7328), while completing training in a reasonable time of 2.44 seconds. These results indicate that XGB effectively balances model complexity and generalization, making it a reliable choice for this dataset. LR, RF, ANN, KNN delivered comparable performances by presenting a balanced trade-off between speed and accuracy, making them suitable for applications with moderate accuracy requirements and faster training needs. However, DNN and H2ODeepLearning (H_SOD) models struggled to achieve comparable performance, with training accuracies of 0.6105 and 0.4040, respectively. These models also required significantly longer training times, indicating potential inefficiencies for this particular dataset. Fig 7 provides a visual comparison of these models based on average accuracy, precision, recall, F1-score, and training time. It underscores the relative strengths and weaknesses of each algorithm. While XGBoost demonstrated the most balanced performance, the overall results indicate that the GC dataset might lack the ability to effectively represent underlying patterns, resulting in lower predictive performance across all models compared to the original dataset.

**Table 5:** Performance measures for ML models of synthesized dataset by Gaussian Copula model.

	train	test	SCVTrain	SCVTest	Precision	Recall	F1_Score	time
ANN	0.768	0.76	0.765225	0.7659	0.752789	0.733338	0.739265	8.75865
DNN	0.6105	0.6015	0.604375	0.6065	0.461412	0.496981	0.389262	306.2161
KNN	0.77275	0.7375	0.830125	0.688	0.72561	0.713711	0.717754	0.001341
LR	0.759875	0.762	0.760875	0.7565	0.75834	0.731338	0.738462	0.309331
RF	0.751875	0.7485	0.755625	0.747	0.738688	0.723206	0.728166	1.132361
SVM	0.757125	0.755	0.7567	0.7567	0.750533	0.723537	0.730413	27.85132
XGB	**0.816125**	**0.7515**	0.825375	0.7505	0.74112	0.728403	0.732807	2.442899
H2OXGBoost	**0.923125**	0.733	0.923125	0.733	0.704559	0.713843	0.704754	238.1652
H2ODL^*^	0.404	0.398	0.47675	0.454	0.19575	0.5	0.281351	1815.234

* DL-based Multi Model Ensemble

We did note clear signs of overfitting in the H_OD model, which achieved a high training accuracy (0.9231) but significantly lower testing accuracy (0.7330). This large performance gap suggests the model may have learned noise or specific patterns from the training data that do not generalize well to unseen data. To mitigate overfitting in such high-capacity models, we employed standard techniques including early stopping, tuning regularization parameters and stratified cross-validation. However, we acknowledge that further exploration—such as dropout (in neural networks), ensembling with bagging methods, or data augmentation using more advanced synthetic techniques—could improve generalization.

**Fig 8:**
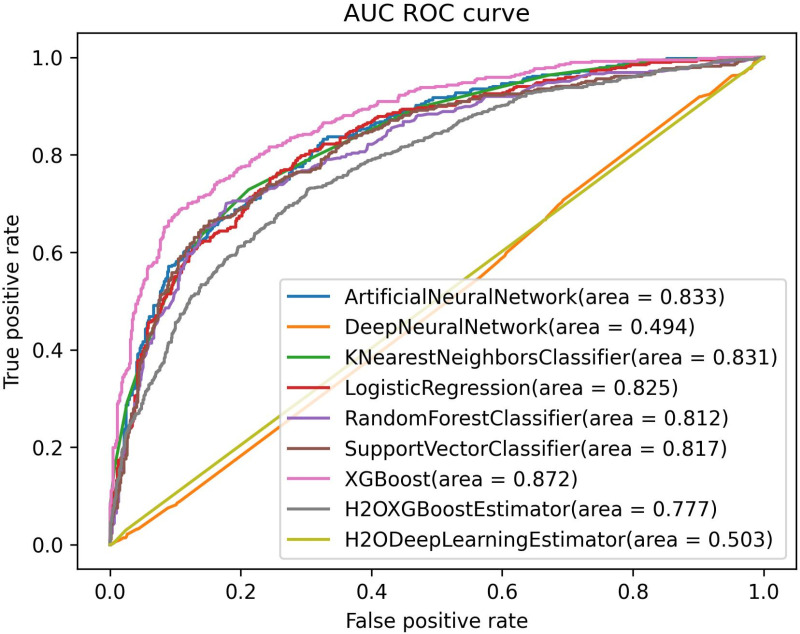
AUC ROC curves of all models for Gaussian Copula model synthesized dataset.

In Fig 8, the XGBoost model stands out as the top performer, achieving the highest AUC of 0.872, indicating superior discrimination capability. ANN and KNN also demonstrated strong classification performance with AUC values of 0.833 and 0.831, respectively, closely trailing the leading model. Meanwhile, LR and SVM performed well, achieving AUC scores of 0.825 and 0.817, respectively, reflecting reliable generalization across different thresholds. RF followed with a slightly lower AUC of 0.812, indicating robust but somewhat less precise classification. In contrast, the H2OXGBoostEstimator and H2ODeepLearningEstimator models struggled, with AUC values of 0.777 and 0.498, respectively, suggesting challenges in capturing the underlying data patterns. The DNN model also recorded a lower AUC of 0.485, reflecting significant limitations in its ability to distinguish between the two classes.

#### Evaluation of Phase 3: Prediction model based on the synthetic data created TVAE.

**Fig 9:**
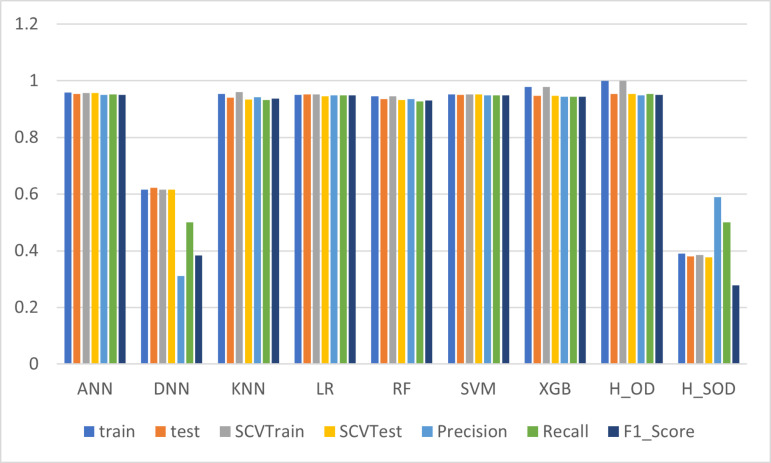
Comparison of Model Performance Metrics of TVAE dataset.

To the phase 3 The Table 6 presents the performance like the Tables 2 and 3 but here the data set is TVAE Dataset. Among the models, H2OXGBoost stands out as the best performer, achieving perfect training accuracy (1.0) and a high testing accuracy (0.953). It also demonstrates strong precision (0.9480), recall (0.9529), and F1-score (0.9503), reflecting reliable predictive power. Despite its longer training time (283.83 seconds), this model outperforms others in overall classification metrics. In comparison, ANN also performed well, achieving a testing accuracy of 0.9535 with similarly high precision (0.9495), recall (0.9527), and F1-score (0.9510), but with a significantly faster training time (7.77 seconds). Furthermore, we can see the visual comparison of all model in Fig 9 and here it is seen that H20XGBoost performs better than other models.

**Table 6:** Performance measures for ML models of synthesized dataset by TVAE.

Model	train	test	SCVTrain	SCVTest	Precision	Recall	F1_Score	time
ANN	**0.959**	**0.9535**	0.95735	0.9564	0.949463	0.952678	0.951009	7.768411
DNN	0.615125	0.6225	0.616625	0.6165	0.31125	0.5	0.383667	260.1538
KNN	0.95425	0.941	0.9605	0.9335	0.942421	0.932439	0.936931	0.001838
LR	0.9505	0.952	0.95175	0.945	0.949443	0.948998	0.949219	0.04356
RF	0.944625	0.935	0.944875	0.932	0.934651	0.927327	0.930704	0.992389
SVM	0.951625	0.951	0.9515	0.9515	0.947805	0.94868	0.948238	14.63533
XGB	0.979	0.947	0.977625	0.9465	0.944348	0.943465	0.943902	2.063429
H2OXGBoost	**1**	0.953	1	0.953	0.947959	0.952866	0.950277	283.8304
H2ODL^*^	0.3895	0.38	0.384875	0.3775	0.588972	0.500944	0.277382	1500.974

* DL-based Multi Model Ensemble

**Fig 10:**
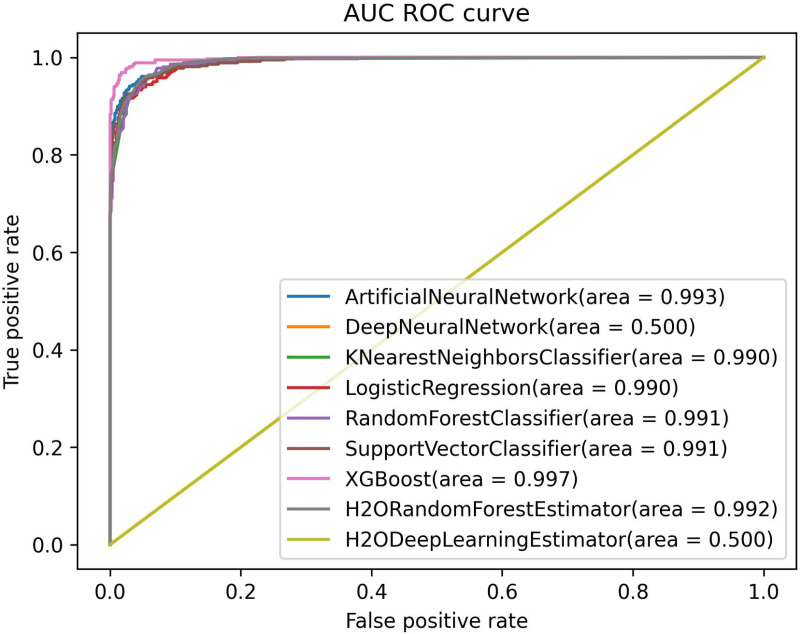
AUC ROC curves of all models for TVAE dataset.

Furthermore, Fig 10 shows the AUC-ROC curves of the models, where the AUC represents the model’s performance. In this case, ANN has the highest AUC value of 0.994, indicating its superior ability to distinguish between the classes. Additionally, it is noteworthy that the models, including KNN, LR, RF, SVM, and H_OD, also demonstrate strong performance with high AUC values of 0.990 to 0.992. Overall, the high AUC values for most models suggest effective generalization on the dataset, indicating their ability to reliably classify and predict outcomes.

## Discussion

### Finding the best model from each phase

Table 7 presents a summary of the performance of the top two models selected from each of the three datasets. In Phase 1, which uses the original dataset, both the KNN and LR models demonstrate high accuracy, with KNN achieving a training accuracy of 0.9824 and a testing accuracy of 0.9737, while LR achieves a training accuracy of 0.9846 and a testing accuracy of 0.9737. The robust precision, recall, and F1_score of these models highlight their reliability in classification tasks. For Phase 2, synthesized data is derived from the GC dataset. In this phase, the H2OXGBoost model achieves a training accuracy of 0.9231 and a testing accuracy of 0.7330 (Overfitted), while the XGB model records a training accuracy of 0.8161 and a testing accuracy of 0.7515, reflecting slightly better performance in testing. These performances trail those achieved with the original dataset, illustrating the complexities of the synthesized data. Finally, in Phase 3, where the data is synthesized via TVAE, the H2OXGBoost model excels with a flawless training accuracy of 1.0000 and a testing accuracy of 0.9530, demonstrating exceptional classification performance. Another notable performer in this phase is the ANN model, which delivers a consistent training accuracy of 0.9590 and a testing accuracy of 0.9535, reflecting strong generalization to unseen data.

**Table 7:** Best models of Tables 2–4.

Dataset	Model	train	test	SCVTrain	SCVTest	Precision	Recall	F1_Score	time
Orginal	KNN	0.9824	0.9737	0.9803	0.9823	0.9741	0.9692	0.9716	0.0009
	LR	0.9846	0.9737	0.9803	0.9646	0.9697	0.9742	0.9719	0.0058
GC	H_OD	0.9231	0.7330	0.9231	0.7330	0.7046	0.7138	0.7048	238.1652
	XGB	0.8161	0.7515	0.8254	0.7505	0.7411	0.7284	0.7328	2.4429
TVAE	H_OD	1.0000	0.9530	1.0000	0.9530	0.9480	0.9529	0.9503	283.8304
	ANN	0.9590	0.9535	0.9574	0.9564	0.9495	0.9527	0.9510	7.7684

It can be summarized that, TVAE model is better to use for synthesized data generation in the case of Breast Cancer as per overall performance of models on individual dataset. All ML models performs similar in Original and TVAE datasets (Tables 2 and 4) Apart from that, some models performs better than other models for predicting breast cancer. For Original dataset KNN performs better than other models in a very moderate way and for TVAE dataset H2OXGBoost performs better than others with 100% training accuracy.

When considering the ease of use, including the hassle of hyperparameter tuning and data preprocessing, frameworks like H2O AutoML remain advantageous, as they automate these steps, significantly reducing the time and effort required for model development. Given these considerations, H2O AutoML is a highly effective choice for synthetic data-based breast cancer prediction, outperforming many conventional ML models, including the ensemble-based ANN used in this study.

### Comparative study

From our review of the literature, we identified only six studies that utilized the Wisconsin Breast Cancer Diagnostic Dataset, which comprises of 569 instances and 30 features. Four of these studies focused on identifying suitable ML techniques for tumor cell classification, while one study explored the use of AutoML, and another researched the potential application of synthetic data generation methods for tumor cell classification. Our study holds particular significance as we not only applied a DL-based multi-model ensemble—a methodology not previously used on this dataset—but also examined the utility of a novel AutoML framework, called H2O, in classifying tumor cells. To benchmark our results, we collected the highest accuracy values from the ML techniques utilized in the analyzed literature. We then compared these values with the performance of our top two models from each phase of our study.

From the Table 8, we can see that in terms of traditional ML models applied to the original dataset, our study has shown superior accuracy compared to other models presented in various literature. Our study saw KNN achieve an accuracy of 98.24%, outperforming other models. When considering AutoML models, [13] achieved an impressive accuracy of 98.60% with TPOT. However, our study also demonstrated high accuracy with the H_OD model (Original data) with 97.36%. These results show that both manual and automated ML methods can achieve high performance in breast cancer prediction tasks. For synthetic data generated by TVAE, our study also compared favorably. Although the TabNet model in the study by Inan et al. [31] achieved an accuracy of 96.66% and XGBoost reached 94.90% accuracy, our study outperformed these results with H2OXGBoost reaching perfect 100% accuracy in training set and 95.3% in testing set. Overall, the high accuracy achieved by various models in our study indicates the viability of both traditional ML models and AutoML for breast cancer prediction using original or synthetic data. It also reinforces the potential of synthetic data, produced by methods like TVAE, in this field.

**Table 8:** Comparison of accuracies with other authors.

	Author	Method	Accuracy
Original Dataset and ML models	[14]	SVM	92.70%
	[15]	Simple Logistic Method	75.17%
	[12]	Naïve Bayes	87.41%
	[11]	RF	96.50%
	**Our Study**	**KNN**	**98.24%**
	**Our Study**	**LR**	**98.46%**
Use of AutoML	[13]	Auto-Sklearn	97.50%
	[13]	**TPOT**	**98.60%**
	**Our Study (Original data)**	H2OXGBoost	97.36%
TVAE generated synthetic data	[36]	**Tabnet**	**96.66%**
	[36]	XgBoost	94.90%
	**Our Study**	**H2ODRF**	**100%**

## Conclusions

This study’s objective was to analyze and contrast the effectiveness of distinct ML models, either standalone or in conjunction with a DNN, along with the application of AutoML for the purpose of predicting breast cancer. The research also probed the enhancement of prediction performance using synthetic data produced via TVAE and Gaussian Copula. Furthermore, a 5-fold stratified cross-validation technique was employed to mitigate biases and challenges related to overfitting.

Firstly, the study demonstrated the effectiveness of different ML models, including traditional models such as KNN, SVM, ANN, and RF, as well as ensemble models like XGBoost and AutoML, in accurately predicting breast cancer. These models achieved high levels of accuracy, with KNN reaching an impressive accuracy of 98.24% on the original dataset. The findings highlight the viability of both traditional ML models and AutoML in breast cancer prediction tasks. Furthermore, the research explored the potential of DL-based ensemble strategies and AutoML-based ensemble strategies to further improve prediction accuracy. The use of multi-model ensembles, particularly those incorporating DNN and AutoML, showed promising results and demonstrated the power of combining multiple models for enhanced performance.

In addition to evaluating different ML models, the study investigated the potential of synthetic data generation techniques, specifically Gaussian Copula and TVAE, for data augmentation. The outcomes showed that the application of the synthetic data created by TVAE resulted in increased accuracy, with the H2OXGBoost model registering an accuracy rate of 100% in train and 95.3% in test. This underlines the significance of synthetic data in boosting the efficiency of machine-learning models in predicting breast cancer.

On the other side, the AutoML framework proved to be highly effective in our research due to its ability to automate the model creation and hyperparameter optimization process. This automation allowed us to streamline and expedite the model development phase, resulting in improved model performance and scalability additionally AutoML is easy to apply and modify. By leveraging advanced techniques and ensemble strategies, AutoML efficiently evaluated and compared a wide range of ML models, ultimately selecting the best-performing model based on key evaluation metrics such as accuracy and AUC. The utilization of AutoML, played a crucial role in augmenting traditional ML approaches in our research, ensuring that we achieved high prediction accuracy without falling into the pitfalls of overfitting or underfitting the data. Despite its advantages, AutoML also has limitations. The automated process of model creation and hyperparameter optimization can be time-consuming, especially when dealing with large datasets or complex models. This can hinder decision-making when immediate results are required.

This research paper offers important insights into the accuracy and performance of various machine learning models, ensemble strategies, and synthetic data generation techniques for breast cancer prediction. However, several limitations should be acknowledged.

- First, the study relied on a relatively small real-world dataset (569 instances), which may constrain the generalizability of the findings.
- Second, while synthetic data techniques such as Gaussian Copula and TVAE helped address data scarcity, they lagged a bit to replicate the complexity of real clinical distributions, especially in edge cases.
- Third, the scope of features was limited to structured clinical data; incorporating imaging and genomic data could potentially improve predictive accuracy.
- Additionally, we observed that certain models, such as H2ODL, had significantly higher training time requirements and poor performance, which may hinder practical deployment in resource-constrained settings.

Despite these limitations, the findings contribute meaningfully to breast cancer prediction research. The study addresses critical gaps such as comparing AutoML with traditional methods, evaluating synthetic data generation, and exploring DL-based ensemble strategies. The chosen research methodologies ensured reliability and reproducibility of results. The insights gained here can aid in enhancing diagnostic models and guiding treatment decision-making. Future research could explore more advanced deep learning-based multi-model ensemble approaches, incorporate multimodal data (including imaging), and validate models on larger, more diverse datasets to further boost performance and applicability in real-world healthcare scenarios. This work holds substantial promise for both medical professionals and ML practitioners, especially in generating training data where real medical datasets are limited.
